# KCa3.1 ion channel: A novel therapeutic target for corneal fibrosis

**DOI:** 10.1371/journal.pone.0192145

**Published:** 2018-03-19

**Authors:** Govindaraj Anumanthan, Suneel Gupta, Michael K. Fink, Nathan P. Hesemann, Douglas K. Bowles, Lindsey M. McDaniel, Maaz Muhammad, Rajiv R. Mohan

**Affiliations:** 1 Harry S. Truman Memorial Veteran Hospital, Columbia, Missouri, United States of America; 2 Veterinary Medicine and Surgery, University of Missouri, Columbia, Missouri, United States of America; 3 Mason Eye Institute, University of Missouri School of Medicine, Columbia, Missouri, United States of America; 4 Biomedical Sciences, College of Veterinary Medicine, University of Missouri, Columbia, Missouri, United States of America; Cedars-Sinai Medical Center, UNITED STATES

## Abstract

Vision impairment from corneal fibrosis is a common consequence of irregular corneal wound healing after injury. Intermediate-conductance calmodulin/calcium-activated K^+^ channels 3.1 (KCa3.1) play an important role in cell cycle progression and cellular proliferation. Proliferation and differentiation of corneal fibroblasts to myofibroblasts can lead to corneal fibrosis after injury. KCa3.1 has been shown in many non-ocular tissues to promote fibrosis, but its role in corneal fibrosis is still unknown. In this study, we characterized the expression KCa3.1 in the human cornea and its role in corneal wound healing *in vivo* using a KCa3.1 knockout (KCa3.1^-/-^) mouse model. Additionally, we tested the hypothesis that blockade of KCa3.1 by a selective KCa3.1 inhibitor, TRAM-34, could augment a novel interventional approach for controlling corneal fibrosis in our established *in vitro* model of corneal fibrosis. The expression of KCa3.1 gene and protein was analyzed in human and murine corneas. Primary human corneal fibroblast (HCF) cultures were used to examine the potential of TRAM-34 in treating corneal fibrosis by measuring levels of pro-fibrotic genes, proteins, and cellular migration using real-time quantitative qPCR, Western blotting, and scratch assay, respectively. Cytotoxicity of TRAM-34 was tested with trypan blue assay, and pro-fibrotic marker expression was tested in KCa3.1^-/-^. Expression of KCa3.1 mRNA and protein was detected in all three layers of the human cornea. The KCa3.1^-/-^ mice demonstrated significantly reduced corneal fibrosis and expression of pro-fibrotic marker genes such as collagen I and α-smooth muscle actin (α-SMA), suggesting that KCa3.1 plays an important role corneal wound healing *in vivo*. Pharmacological treatment with TRAM-34 significantly attenuated corneal fibrosis *in vitro*, as demonstrated in HCFs by the inhibition TGFβ-mediated transcription of pro-fibrotic collagen I mRNA and α-SMA mRNA and protein expression (p<0.001). No evidence of cytotoxicity was observed. Our study suggests that KCa3.1 regulates corneal wound healing and that blockade of KCa3.1 by TRAM-34 offers a potential therapeutic strategy for developing therapies to cure corneal fibrosis *in vivo*.

## Introduction

Corneal scarring from traumatic or infectious injury is a major cause of vision impairment worldwide [1]. While various forms of surgical keratoplasty are used to treat visually significant corneal scarring, such surgery is not without drawbacks. Surgical keratoplasty requires specialized technical skills, may be associated with further complications and often requires life-long monitoring and follow-up by cornea specialists [2]. The pharmacological options available to reduce corneal fibrosis also have drawbacks, including the risk of a variety of undesired side effects.

Aberrant corneal wound healing after ocular injury typically involves fibrosis and a non-functional mass of fibrotic tissue [3]. Corneal healing after injury or surgery, including keratoplasty and LASIK, is a complex process that entails increased cytokine expression, keratocyte activation and the myofibroblast formation and enhanced deposition of extracellular matrix (ECM) proteins [4,5]. Studies have shown that the pro-fibrotic cytokines produced by epithelial and inflammatory cells in response to injury lead fibrogenic events [3]. The multi-functional cytokine Transforming growth factor beta (TGFβ) has been associated as having an important role in various fibrotic eye diseases, such as anterior sub-capsular cataract, opacification of corneal and posterior capsules, orbital and sub-macular fibrosis, and glaucoma [6,7].

A common sequel in fibrotic eye diseases is the transdifferentiation of fibroblasts into contractile and secretory myofibroblasts. Myofibroblasts play a necessary and beneficial role in wound healing processes. In pathological condition, myofibroblasts rapidly produce excessive amounts of ECM proteins and exert contraction across the ECM, resulting in the alteration of tissue architecture and impair organ function. TGFβ plays a major role in the process of fibroblast activation, with activated fibroblasts eventually transdifferentiating into myofibroblasts. Alpha-smooth muscle actin (α-SMA) expressing myofibroblasts, with their contractile properties, play a significant role in the formation of fibrosis [8]. Persistence of opaque myofibroblasts in the stroma leads to stromal opacity and corneal haze. Many studies suggest that a reduction in the myofibroblast population directly correlates with a decrease in the severity of corneal fibrosis and an increase in corneal transparency following corneal injury [9,10]. Since TGFβ plays an integral role in myofibroblast formation, attenuation of TGFβ signaling is a potential strategy to prevent corneal fibrosis.

Intermediate-conductance calmodulin/calcium-activated K^+^ channels 3.1 (KCa3.1) (also known as IK1, encoded by the KCNN4 gene) proteins are expressed in mitochondrial and cytoplasmic membranes [11]. KCa3.1 is known to regulate cell cycle progression and proliferation. KCa3.1 proteins are activated several folds in response to injury [12]. The literature also points to the role of KCa3.1 in the development of fibrotic disorders of the lung, kidney and liver [13–15]. In murine renal fibroblasts, mitogenic stimulation was found to upregulate expression of KCa3.1 several folds [12]. In addition, a dramatic upregulation of KCa3.1 protein in renal fibroblasts in response to injury was accompanied by increased expression of fibroblast-specific protein 1, collagen I, collagen III and TGFβ [12]. However, to date, the expression of KCa3.1 in the corneal stroma has not been studied, nor has the ability of the selective KCa3.1 inhibitor TRAM-34 to block corneal fibrosis. It is our hypothesis that the blockade of KCa3.1 with selective inhibitors could offer a potential therapeutic strategy for TGFβ-induced corneal fibrosis.

We and others have shown that activation and proliferation of corneal keratocytes and fibroblasts in the corneal stroma are triggered by locally secreted pro-fibrotic cytokines, including TGFβ. Agents such as decorin, bone morphogenic protein 7, Smad7, pirfenidone, mitomycin C, trichostatin A, and vorinostat have been demonstrated to suppress fibroblast proliferation and inhibit corneal fibrosis *in vivo* [10,16–20]. The accumulating literature reveals that KCa3.1 ion channels can significantly increase cellular proliferation by increasing intracellular Ca^++^ signaling and altering cell cycle progression [21,22]. Thus, in this study we investigated the expression of KCa3.1 protein in the cornea and its role in corneal wound healing modulation in donor human cornea and in KCa3.1^-/-^ mice. Furthermore, we investigated whether the blockade of KCa3.1 ion channel function has the potential to offer a novel interventional strategy to treat corneal fibrosis *in vivo* without major cytotoxicity.

## Materials and methods

### *In vivo* animal and *ex vivo* donor human corneas

All experiments on human corneal tissue were carried out in accordance with the tenets of the Declaration of Helsinki and the ethical principles for medical research involving human tissues, as well as in accordance with the rules and regulations of the Institutional Review Board of the University of Missouri. Healthy transparent human corneas were purchased from the Saving Sight, Kansas City, Missouri, and handled as described previously [20]. Twelve healthy corneas from male and female donors (23–78 years of age) within 10 days of death were used to generate primary HSF cultures. Additional, six healthy donor human corneas were utilized for histological studies.

All animals were treated in accordance with the Association of Research in Vision and Ophthalmology (ARVO) Statement for the Use of Animals in Ophthalmic and Vision Research, and the protocol was approved by the University of Missouri Institutional Animal Care and Use Committee as described previously [23]. KCa3.1^-/-^ mice were obtained from Jackson Laboratory (stock #018826); the genetic background is C57BL/J6 × 129, as previously described [11]. KCa3.1^-/-^ mice were bred by homozygote breeding and KCa3.1 gene deficiency was confirmed by quantitative polymerase chain reaction (qPCR). Corneal injury was performed in wild-type (WT) and KCa3.1^-/-^ mice by placing a 2-mm diameter filter paper disc, presoaked in 0.5 N NaOH, on the central cornea for 30 seconds, followed by extensive rinsing with balanced salt solution (Alcon, Fort Worth, TX), as previously described [24]. Alkali burn was created at day 0 and animals were euthanized 3 or 7 days post-injury.

### Primary human corneal fibroblast (HCF) cultures

HCF cultures were generated from donor human corneas. Donor corneas were washed with sterile minimal essential medium (MEM; Gibco, Grand Island, NY), and corneal epithelial and endothelium layers were gently removed with a surgical blade. The remaining stromal tissues were cut into small pieces, placed on a culture dish, and incubated in a humidified 5% CO_2_ incubator at 37 °C in Dulbecco’s modified Eagle’s medium (DMEM), supplemented with 10% fetal bovine serum (FBS), for 2–4 weeks to obtain HCF cultures. To obtain fibroblast cultures, these primary cells were harvested from corneal buttons, and grown in 6-well plates, at an initial density of 7.5 x 10^4^, in DMEM supplemented with 10% FBS.

### Recombinant human TGFβ1 and TRAM-34 treatment

HCFs were seeded at a density of 7.5 × 10^4^ in DMEM medium containing 10% fetal bovine serum and treated with either recombinant human TGFβ1 (rhTGFβ1, 5 ng/ml; PeproTech, Rocky Hills, NJ) or TRAM-34 (25 μM; Tocris Biosciences, Bristol, UK) or the combination of rhTGFβ1 (5 ng/ml) and TRAM-34 (25 μM) for 24 hours and 72 hours. Transformation of corneal fibroblasts to myofibroblasts is known to occur when HCFs are grown under serum-free conditions in TGFβ1 (5 ng/ml) alone for 4 days [9]. Cultures were washed twice on with cold 1X PBS. mRNA was isolated and converted into cDNA for quantitative real-time polymerase chain react (qRT-PCR) analysis. Each experiment was performed in triplicate.

### Quantitative real-time PCR

Total mRNA was isolated using the RNeasy kit (Qiagen, Valencia, CA), according to the manufacturer’s protocol. First-strand cDNA was synthesized by reverse transcriptase enzyme (Promega, Madison, WI). qRT-PCR was performed using the One Step Plus Real-Time PCR system (Applied Biosystems, Carlsbad, CA). Table 1 lists the gene-specific forward and reverse primer sequences used in PCR analyses. A 20 μl reaction mixture contained 1 μl cDNA, 2 μl forward primer, 2 μl reverse primer, and 10 μl iQ^™^ SYBR^®^ Green Super mix (Bio-Rad Laboratories, Hercules, CA). The mixture was exposed to the following PCR parameters: 95°C for 5 minutes, followed by 40 cycles of 95°C for 15 seconds, then 60°C for 1 minute, and then a final cycle of 72°C for 10 minutes. The fluorescence threshold value (C_t_) was calculated to detect signal differences in association with an exponential increase of PCR products in the log linear phase. Relative expression/fold change over the corresponding values for the control was calculated by the 2^-ΔΔCt^ method. Two to three independent experiments were executed, and for each sample, qRT-PCR was performed in triplicate and the average fold changes in mRNA levels were calculated.

**Table 1:** PCR primers.

Gene	Protein	Accession	Primers
KCa3.1	Calcium-activated potassium channel 3.1	NM_002250.2	**F**: GCCCTGGAGAAACAGATTGA **R**: CATAGCAGCATAGTGAGAGTG
α-SMA	α-smooth muscle actin	NM_001613	**F**: TGGGTGACGAAGCACAGAGC **R**: CTTCAGGGGCAACACGAAGC
Col1A1	Collagen I	NM_000088.3	**F**: TGTGGCCCAGAAGAACTGGTACAT **R**: ACTGGAATCCATCGGTCATGCTCT
ß-actin	ß-actin	X00351.1	**F**: AGGCCAACCGCGAGAAGATGACC **R**: GAAGTCCAGGGCGACGTAGCAC
α-SMA	α-smooth muscle actin	NM_007392.3	**F**: GGGAGTAATGGTTGGAATGG **R**: GATGATGCCGTGTTCTATCG
Col1A1	Collagen I	NM_007742.4	**F**: CGGTTATGACTTCAGCTTCC **R**: CGAACCACGTTAGCATCATC
Col4A1	Collagen IV	NM_009931.2	**F**: GGCATTGTGGAGTGTCAA **R**: CCTTTCTGACCTTTCTGTCC
TGFβ1	Transforming growth factor-β1	NM_011577	**F**: CTACCATGCCAACTTCTGTC **R**: GGGTTGTGTTGGTTGTAGAG
ß-actin	ß-actin	NM_007393.3	**F**: TGGCTACAGCTTCACCACCA **R**: GAAGAGCTATGAGCTGCCTG

For conventional PCR, 50 μl reaction mixtures contained 10 μl buffer (5x green GoTaq^®^ Flexi Buffer; Promega, Madison, WI), 8 μl MgCl_2_ (Promega), 1 μl dNTP mix (Promega), 1 μl forward and 1 μl reverse primers (0.4 μM each), 0.25 μl GoTaq^®^Flexi DNA (Promega), 26.75 μl DEPC treated water and 2 μl of cDNA. Cycle details were as follows: 95°C for 2 minutes, followed by 40 cycles each at 95°C for 30 seconds, then 50°C for 30 seconds, then 72°C for 1 minute, and finally at 72°C for 10 minutes. Beta actin (β-actin) was used as a housekeeping gene.

### Trypan blue exclusion assay

The cytotoxicity of TRAM-34 was evaluated by performing a trypan blue exclusion test. Viable cells were counted *in vitro*, according to manufacturer’s instructions. Briefly, 3 ×10^4^ HCFs were plated in a 12-well tissue culture plate (Thermo Fisher Scientific, Waltham, MA), grown for 24 hours, and then treated with TRAM-34 in various doses or no TRAM-34 (0, 0.5, 1, 5, 10, 25 μM) for 24 hours. For assessment of time-dependent cytotoxicity, HCFs were incubated with TRAM-34 (25 μM) for up to 7 days. After staining with trypan blue (25-900-Cl; Mediatech, Manassas, VA) per the vendor’s instructions, the dye-stained and the dye-unstained cells were counted in a hemocytometer under microscope. The cellular viability percentage was calculated by applying the following formula mentioned in Eq 1. Three independent experiments were performed.

$$\%\;cell\;viability=(\frac{viable\;cell\;count\;}{total\;cell\;count})X\;100\%.$$ (1)

### Cellular migration

The effects of TRAM-34 on TGFβ induced migration in HCFs were examined using an *in vitro* scratch assay, as described earlier [25]. HCFs were seeded in a 6-well plate with MEM supplemented with 10% FBS and incubated overnight. Once HCFs reached 70% confluence, an identical gap was created on each plate with a p200 pipet tip. Cultures were washed with PBS to remove cellular debris and grown with or without rhTGFβ1 (5 ng/ml) in the presence or absence of TRAM-34 (25 μM) for 24 hours. Images were collected at selected time points using a phase-contrast microscope equipped with a Leica DFC290 imaging system (Leica Microsystems, Bannockburn, IL). Comparative analyses of images from different time points and treatment groups were performed.

### Western blot analyses

HCFs were seeded at an initial density of 7.5 × 10^4^ using medium supplemented with 10% serum. The mixture was switched to serum-free medium when HCFs reached 50% confluence. Cultures at 70% confluence were treated for 48 hours with either vehicle, rhTGFβ1 (5 ng/ml), TRAM-34 (25 μM) or the combination of rhTGFβ1 and TRAM-34. Protein lysates were prepared and quantified by Bradford assay, as described previously [20]. Samples were resolved on 4–12% sodium dodecyl sulfate (SDS) polyacrylamide gel, transferred onto polyvinylidene fluoride membrane, incubated with β-actin antibodies (Santa Cruz Biotechnology, Santa Cruz, CA) and α-SMA antibodies (Abcam, Cambridge, MA), followed by application of alkaline phosphatase-conjugated anti-mouse secondary antibodies and Nitro-blue tetrazolium chloride and 5-bromo-4-chloro-3’-indolyphosphate p-toluidine (NBT-BCIP) developing reagents. The digital quantification of Western blots was performed using Image Studio software (Version 5.2; Lincoln, NE).

### Immunofluorescence staining

Normal human corneas were embedded in optimal cutting temperature (OCT) compound, cryo-frozen, and sectioned at 7 μm thickness using a Microm HM 525 cryostat (Thermo Fisher Scientific, Waltham, MA). Tissue sections were kept for 10 minutes at room temperature, rinsed in 1X PBS for 5 minutes, outlined with a PAP pen, and blocked using 5% normal donkey serum (Jackson ImmunoResearch Laboratories, West Grove, PA). Tissue sections were then subjected to single immunostaining using anti-IK1 (1:50 dilution, sc-27080; Santa Cruz Biotechnology, Santa Cruz, CA). The mixture was incubated in donkey anti-goat secondary antibody Alexa Fluor^®^ 594 (1:1000 dilution, A11058; Invitrogen, Eugene, OR) for 1 hour at room temperature. For HCFs, cells were fixed with ice-cold methanol for 15 minutes and then washed and blocked for 1 hour at room temperature with 5% normal donkey serum (Jackson ImmunoResearch Laboratories, West Grove, PA) and 0.1% Tween-20 (Sigma-Aldrich, St Louis, MO), followed by a 90-minute application of mouse monoclonal α-SMA antibody (1:200 dilution, M0851; Dako, Carpinteria, CA). Cells were rinsed and then incubated in Alexa Fluor^®^ 488 donkey anti-mouse antibody (1:1000 dilution, A21202; Invitrogen, Eugene, OR) for 1 hour at room temperature. Both the cells and the corneal sections were washed three times in PBS, mounted in Vectashield containing 4’-6-diamidino-2-phenylindole (DAPI, H-1200; Vector Laboratories, Burlington, CA), and photographed with a Leica DM 4000B fluorescent microscope (Leica, Bannockburn, IL) equipped with a digital camera (SpotCam RT KE; Diagnostic Instruments, Sterling Heights, MI).

### Quantification and statistical analysis

Quantification of immunocytochemistry data was performed by counting α-SMA- and DAPI-positive stained cells in 10 randomly selected non-overlapping areas at 200X magnification using a Leica DM 4000B fluorescent microscope (Bannockburn, IL). All results are reported as mean ± standard deviation. The digital quantification of Western blots was performed using Image Studio software (Version 5.2; Lincoln, NE). The qRT-PCR data were analyzed using one-way ANOVA. Data was analyzed for one-way ANOVA using GraphPad Prism 6.0 (GraphPad Software, La Jolla, CA), and p<0.05 was considered to be statistically significant.

## Results

### KCa3.1 expression in human cornea

**Fig 1:**
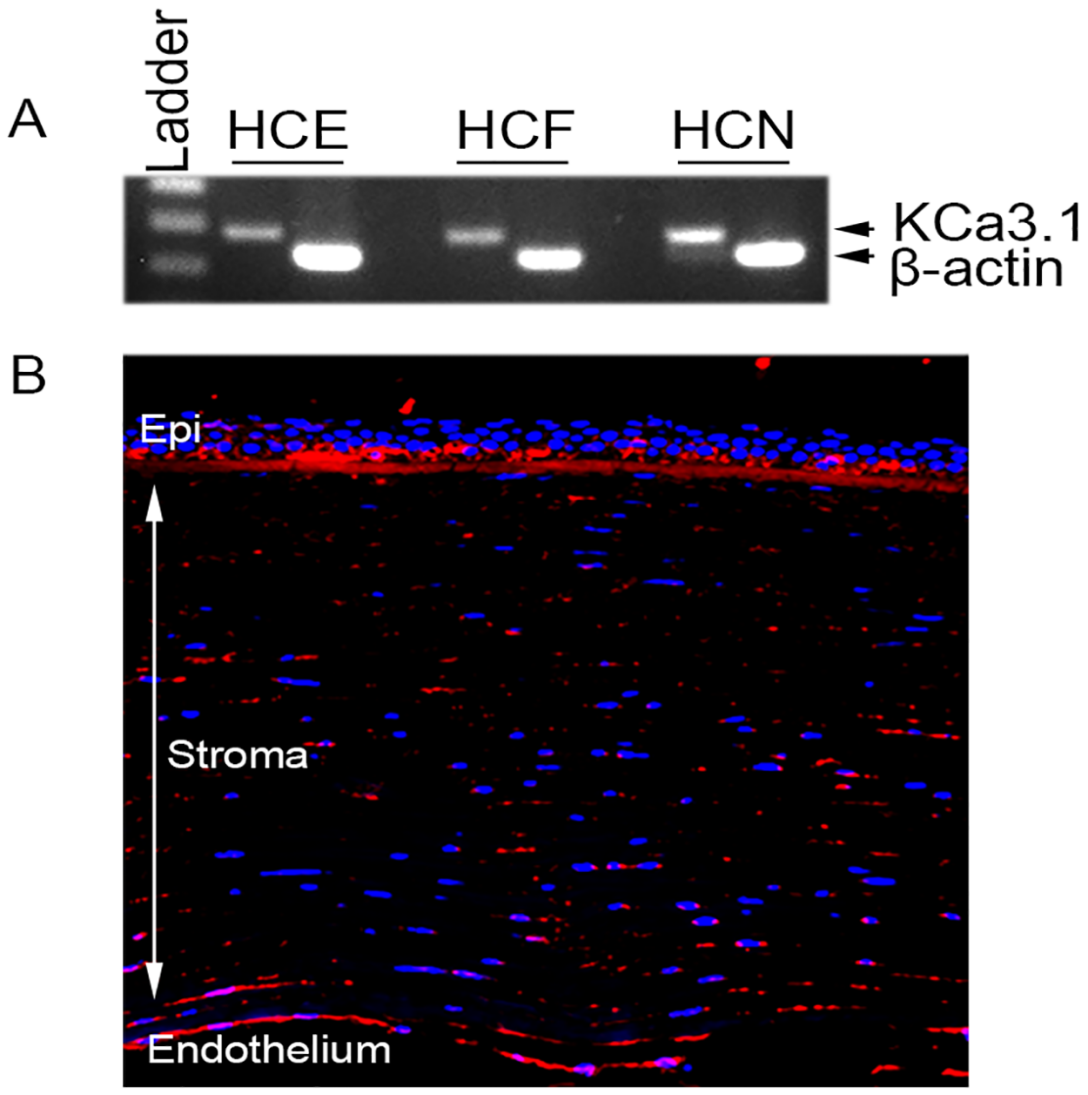
KCa3.1 is expressed in human cornea. **RT-PCR (A) and immunofluorescence (B) showing KCa3.1 expression in normal healthy human cornea**. β-actin was used as internal control. HCE: corneal epithelium, HSF: corneal fibroblast, HCN: corneal endothelium.

RT-PCR and immunofluorescence staining were used to uncover KCa3.1 mRNA and protein expression in various layers of the donor human cornea (Fig 1). The cDNA generated for the human corneal epithelial, stromal and endothelial cells showed substantial KCa3.1 mRNA (Fig 1A). The serial tissue sections of the human donor corneas subjected to immunofluorescence demonstrated substantial KCa3.1 protein (Fig 1B) expression.

### KCa3.1^-/-^ mice show reduced pro-fibrotic gene expression

**Fig 2:**
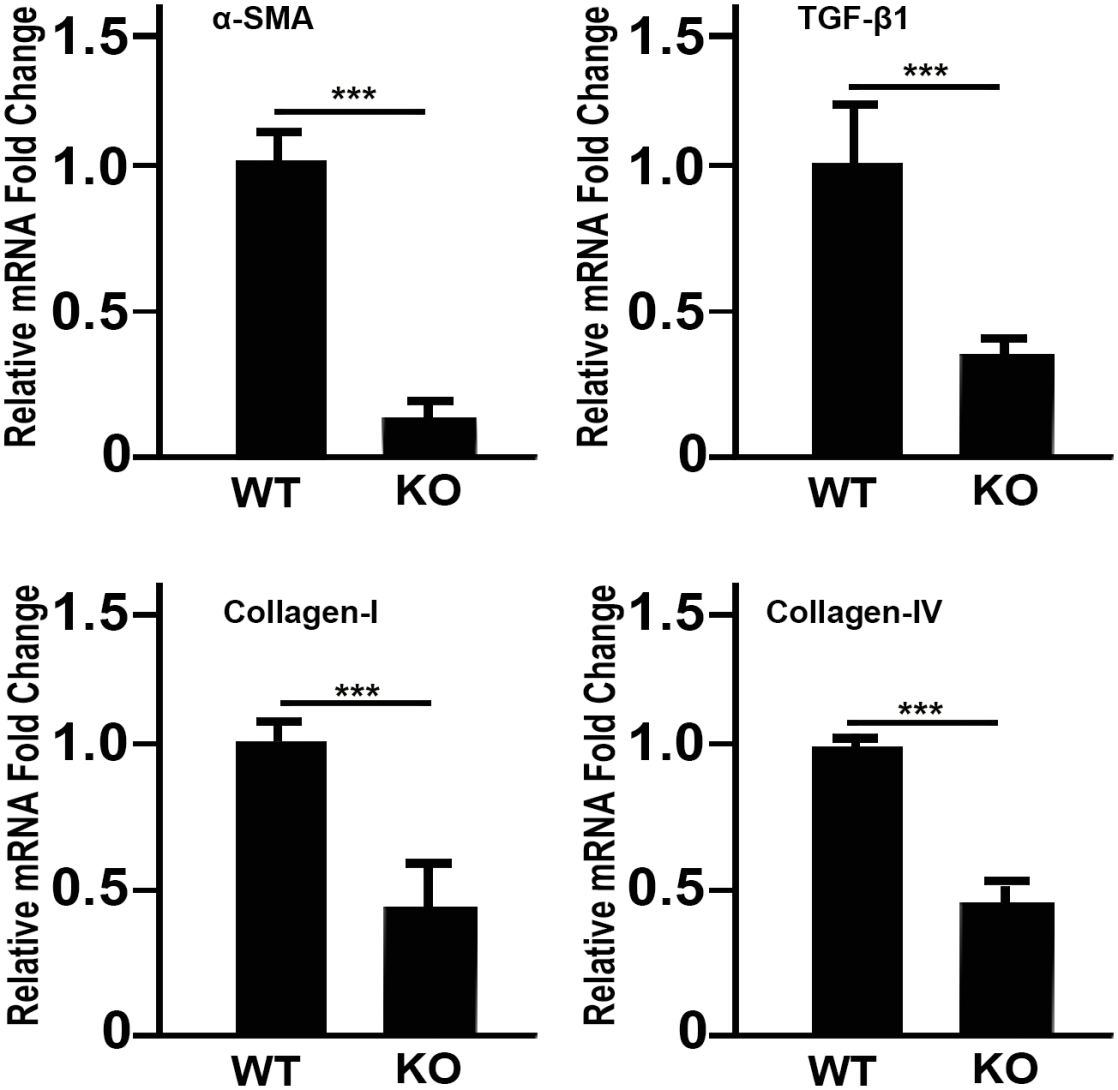
Loss of functional KCa3.1 channels suppresses pro-fibrotic gene expression. Comparison of RT–PCR in WT and KCa3.1^-/-^ mice cornea showed significantly suppressed mRNA expression levels of (A) α-SMA (p<0.001) and (B) collagen I (p<0.001), Collagen IV (p<0.001), and TGFβ1 (p<0.001). Results are expressed as mean ± SEM (*p<0.05, **p<0.01, ***p<0.001).

To test the hypothesis that KCa3.1 deficiency decreases the expression of fibrotic markers in the cornea, the mRNA levels of α-SMA, collagen I, collagen IV and TGFβ1 were quantified in KCa3.1^-/-^ and compared with those in WT mice (Fig 2). The results showed a significant reduction in α-SMA (Fig 2A), collagen I (Fig 2B), collagen IV (Fig 2C) and TGFβ1 (Fig 2D) mRNA expression.

### KCa3.1 deficiency results in reduced fibrosis

**Fig 3:**
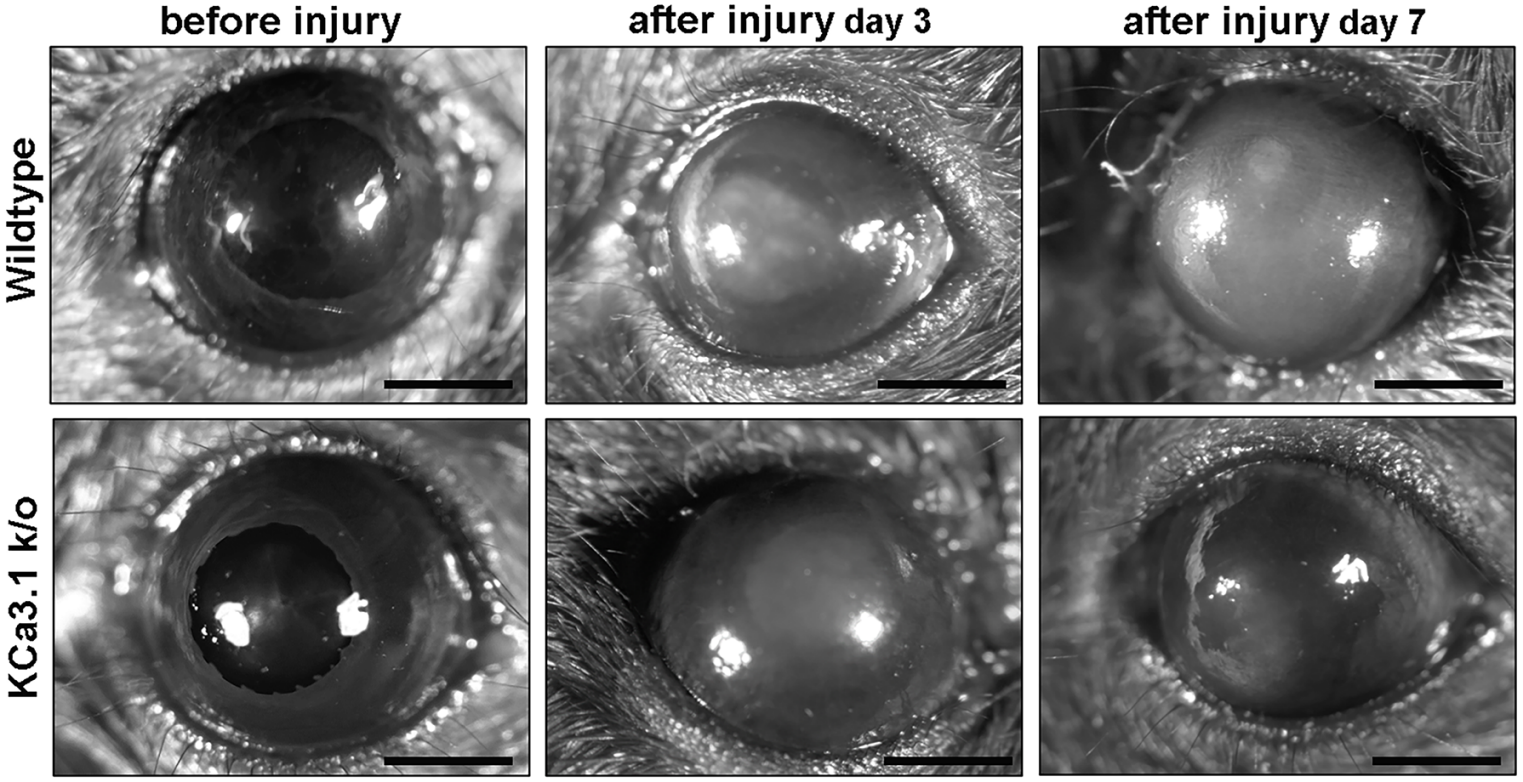
Loss of KCa3.1 reduced corneal haze in mice after alkali wounding. Representative stereomicroscopic images showing corneal haze in Wild type (A, B, C), and KCa3.1^-/-^ mice (D, E, F). Representative examples of Naïve (A, D), 3 days (B, E) and 7 days (C, F) alkali wounding are shown. A decrease in corneal haze was observed in KCa3.1^-/-^ mice.

**Fig 4:**
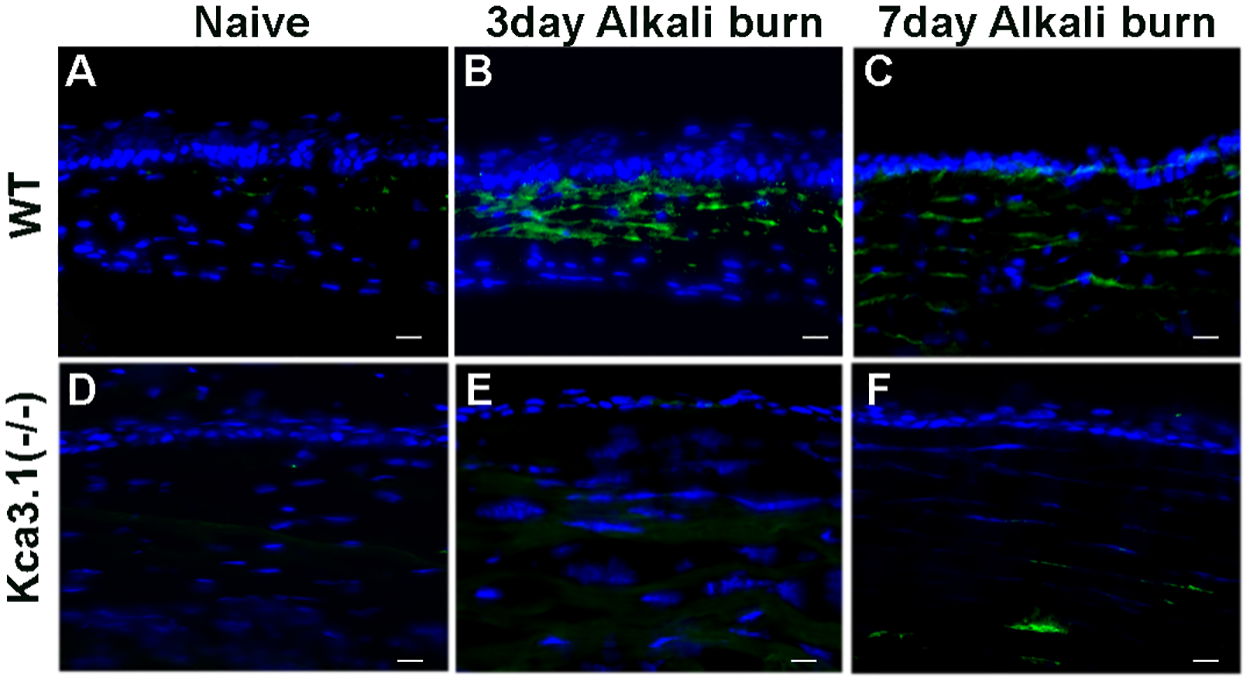
Effect of KCa3.1 loss on corneal fibrosis. Corneal tissue immunostaining of mouse cornea showing levels of α-SMA expression in WT mice at 0, 3 and 7 days (A-C) and KCa3.1^-/-^ mice at 0, 3 and 7 days (D-F) in alkali-induced corneal fibrosis. *Blue*: DAPI-stained nuclei and *green*: α-SMA staining. KCa3.1^-/-^ mouse corneas showed a decrease in α-SMA expression in the stroma compared with that in control WT corneas.

Age- and sex-matched KCa3.1^-/-^ and WT mice were subjected to alkali injury of the cornea to study the role of the KCa3.1 channel in corneal wound healing and fibrosis modulation *in vivo*. The level of corneal opacification in live animals (Fig 3) and the myofibroblast cell level in corneal tissues (Fig 4) were determined at various times after injury with stereo-microscopic examination and immunofluorescence, respectively.

α-SMA expression in response to corneal injury in WT mice is shown in Fig 4A–4C for baseline, 3 and 7 days after alkali injured cornea. Corresponding α-SMA staining in KCa3.1^-/-^ mice is shown in Fig 4D–4F respectively. WT mouse cornea subjected to alkali injury showed an increase in α-SMA staining at 3 days, whereas KCa3.1^-/-^ mouse cornea showed a strikingly decrease in α-SMA -positive stained cells after alkali injury. Collectively, the data provide substantial evidence that the loss of KCa3.1 channel function reverses corneal fibrosis and suggest that pharmacological inhibition of KCa3.1 might be a therapeutic strategy for preventing corneal fibrosis.

### Effect of TRAM-34 on human corneal fibroblast cell viability

**Fig 5:**
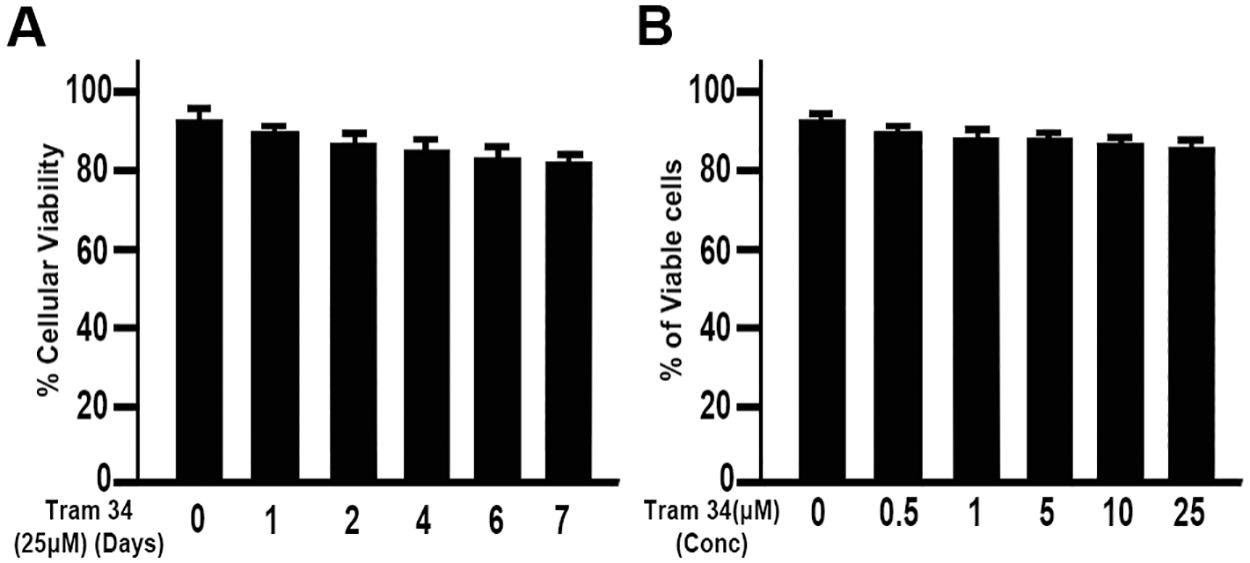
Effect of TRAM-34 on HCF cell viability. No significant difference in cell viability was noted between time points in the controls. A. Cell viability in TRAM-34 exposed cultures up to 7 days of continuous treatment, showing a minimal reduction after 7 days. Concentration dependent changes in cell viability (B). Results are expressed as mean ± SEM.

The effect of TRAM-34 on HCFs was evaluated by analyzing HCF viability (Fig 5). TRAM-34 exposure did not affect HCF viability. HCFs were found to tolerate a 25 μM dose of TRAM-34 for up to 7 days with minimal toxicity (Fig 5A). Cell viability data showed only a moderate decrease in cell viability at the 25 μM dose (Fig 5B). Hence, this dose of TRAM-34 was used for all subsequent experiments.

### TRAM-34 inhibits TGFβ1-induced fibrotic gene expression

**Fig 6:**
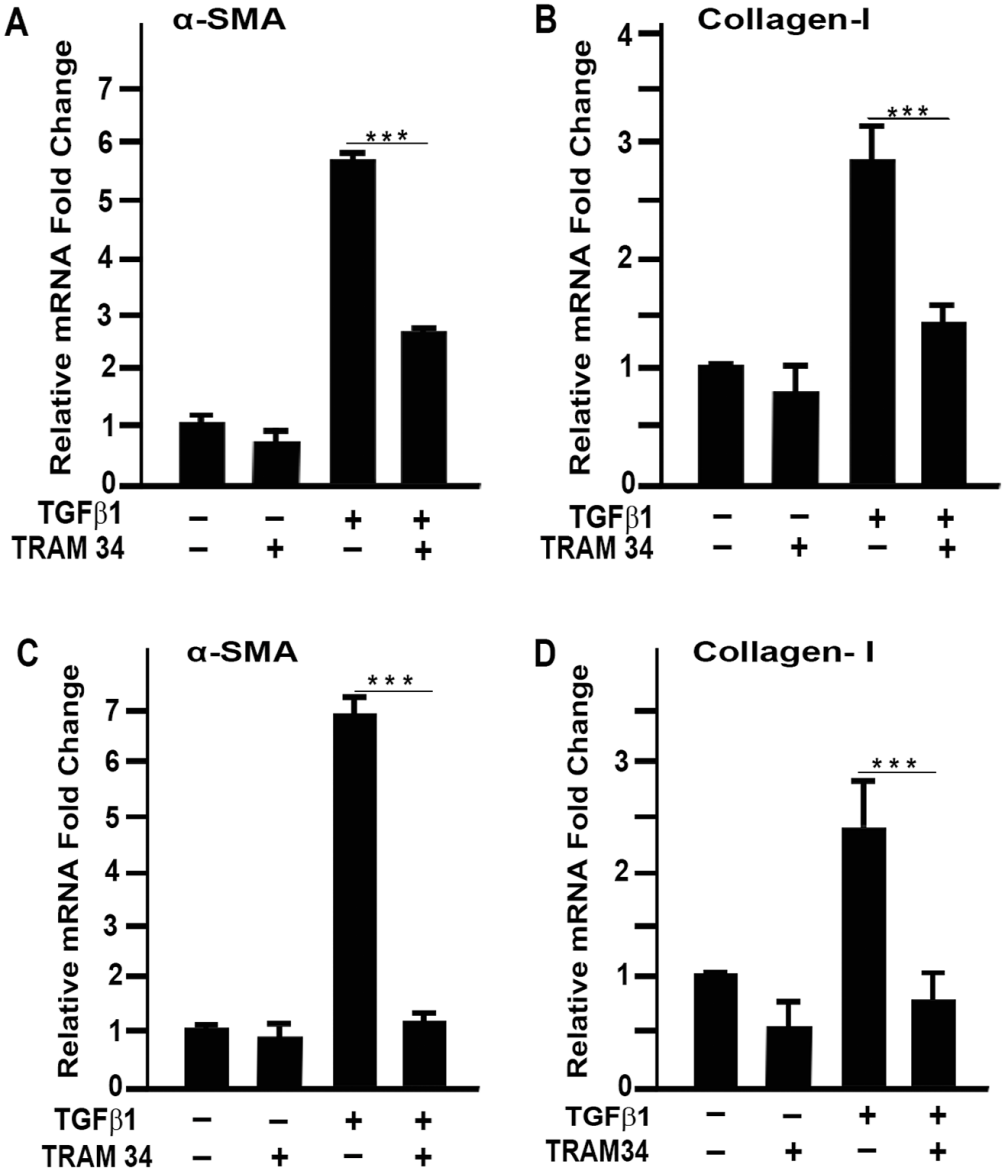
TRAM-34 suppresses TGFβ1 induced pro-fibrotic gene expression. Real-time RT-PCR showed that TRAM-34 treatment in primary human cornea fibroblast cells significantly suppressed TGFβ1 induced mRNA expression levels of (A) α-SMA (p<0.001) and (B) collagen I (p<0.001) at 24 hours (C) and 72 hours (D). Results are expressed as mean ± SEM (*p<0.05, **p<0.01, ***p<0.001).

The *in vivo* experimental results of the blockade of KCa3.1 indicate that KCa3.1 plays a significant role in fibrosis. Studies of the effects of TRAM-34 on the TGFβ1-induced pro-fibrotic gene expression pattern revealed that TRAM-34 treatment significantly decreased pro-fibrotic gene expression (Fig 6). With TGFβ1, α-SMA gene expression increased 5.6 ± 0.2 fold at 24 hours (p<0.001) and 6.8 ± 0.4 fold at 72 hours (p<0.001). TGFβ1 increased collagen I gene expression 2.7 ± 0.5 fold at 24 hours (p<0.01) and 2.3 ± 0.5 fold at 72 hours (p<0.05). TRAM-34 treatment, however, dampened TGFβ1 induced pro-fibrotic gene expression. With TRAM-34 treatment, α-SMA gene expression increased 1.3 ± 0.1 fold at 24 hours (p<0.01) and 5.6 ± 0.2 fold at 72 hours (p<0.001) and collagen I gene expression increased 1.3 ± 0.4 fold at 24 hours (p<0.01) and 1.23 ± 0.4 fold at 72 hours (p<0.01). The dampening of fibrotic gene expression with TRAM-34 is mainly attributed to decreased TGFβ1 induced gene expression.

### α-SMA protein quantification by immunoblotting and immunostaining

**Fig 7:**
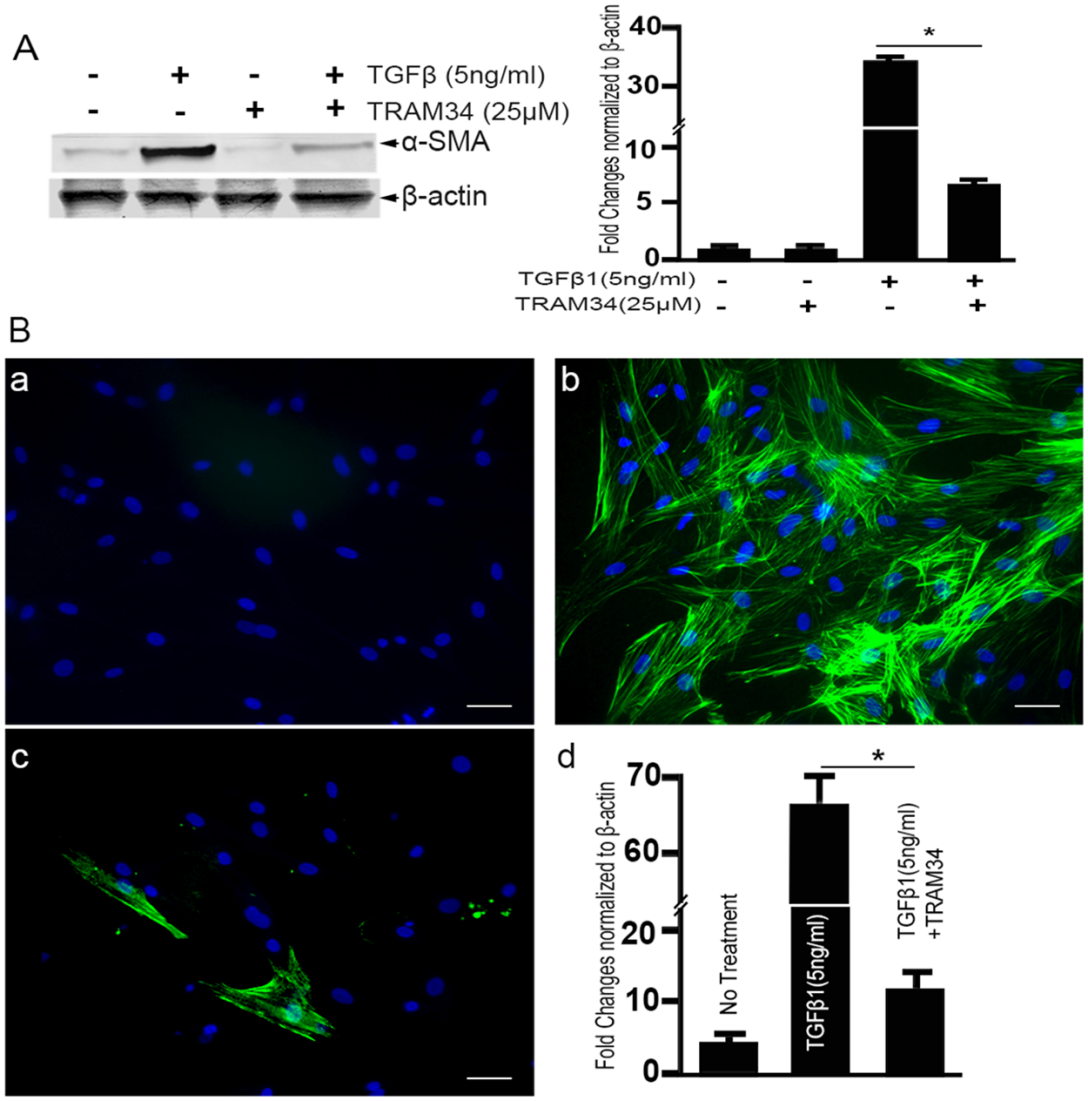
**Western blot analysis (A) and quantification RT-PCR showing the effect of TRAM-34 treatment on α-SMA protein expression**. A prominent α-SMA band was detected in the cellular lysate of TGFβ1 treated HCFs. TRAM-34 treatment attenuated TGFβ1 induced α-SMA expression. Corresponding densitometry analysis of the Western blot is presented in (B). Representative immunostaining images (a, b, c) and their quantification, showing the effect of TRAM-34 treatment on α-SMA protein expression in HCFs. Sparse α-SMA staining (green) can be seen in (a) control (no treatment) cultures compared with cultures grown in the presence of (b) TGFβ1 (+TGFβ1). TRAM-34 (25 μM) +TGFβ1 treatment resulted in a significant decrease in α-SMA (c). Scale bar = 50 μm. The quantitation graph demonstrates a 66% increase in the number of α-SMA positive cells (*p<0.001) when compared with no treatment control, which was significantly attenuated with TRAM34 treatment (p<0.001). (α-SMA = green, DAPI = Blue).

Fibroblast differentiation to α-SMA expressing myofibroblasts is considered a critical step in wound healing. To study the effects of TRAM-34 treatment on TGFβ1 mediated α-SMA protein expression (Fig 7), Western blot analysis (Fig 7A) and immunofluorescence staining (Fig 7B) of α-SMA were performed and demonstrated the TGFβ1 driven transformation of HCFs to myofibroblasts. Western blot analysis of α-SMA protein demonstrated a significant increase (34.6 ± 2.0 fold) in response to rhTGFβ1. While TGFβ1+TRAM-34 treatment was shown to result in a consistent decrease in the magnitude of TGFβ1 mediated α-SMA protein expression (5.5 ±1.0 fold with TRAM-34 versus 34.6 ± 2.0 fold without TRAM-34). Immunofluorescence staining of TGFβ1+TRAM-34 treated HCFs showed fewer α-SMA positive cells than controls (Fig 7A–7C). In contrast, TGFβ1 treatment alone significantly increased α-SMA positive cells compared to controls (Fig 7b). Approximately 5% of the HCFs transformed to myofibroblasts in the absence of the TGFβ1 application, whereas approximately 95% of HCFs transformed to myofibroblasts with TGFβ1 application. Corresponding quantification data of α-SMA immunofluorescence staining is shown in Fig 7D.

### Pharmacological blockade of KCa3.1 limits wound healing

**Fig 8:**
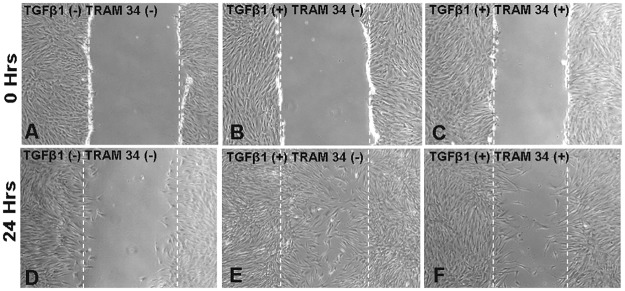
Cell migration scratch assay. The microscopic appearance of the scratch at 0 hours is shown in Figs. A, B and C, and at 24 hours after TRAM-34 treatment in Figs. D, E and F. Composite phase-contrast microscopy image showing HCF taken 24 hours after initiation of treatment with TRAM-34 (25 μM) or TGFβ1 (5 ng/ml). TRAM-34 treatment effectively reduced cellular migration as compared to the TGFβ1 treated control.

KCa3.1 channels play a significant role in wound healing. Whether TRAM-34 can inhibit wound healing events induced by TGFβ1 was evaluated by utilizing a wound scratch assay (Fig 8). An increase in TGFβ1 induced migration of HCFs (Fig 8B–8E) was seen as compared to that in untreated control samples (Fig 8A–8D). The TGFβ1 mediated effect, however, was partially reversed by KCa3.1 channel blockade with TRAM-34 treatment (Fig 8C–8F).

## Discussion

The concurrent increase of KCa3.1 expression with pro-fibrotic genes makes the KCa3.1 channel an attractive therapeutic target. Activation of KCa3.1 channels leads to an influx of Ca^++^. This increase in intracellular calcium stimulates cell cycle progression and proliferation [26]. Recent findings emphasize the significance of blockade of KCa3.1 channel function as a strategy for inhibiting several TGFβ1 dependent cell processes in normal and fibrotic tissues [27–30]. Herein, we provide evidence that the KCa3.1 channel protein is expressed in all three major cellular layers of the human cornea. Further, the findings from our studies indicate that loss of functional KCa3.1 gene expression in mice leads to decreased pro-fibrotic gene expression and reduced expression of the α-SMA expressing myofibroblasts associated with corneal fibrosis. In this study, pharmacological inhibition of KCa3.1 by the selective KCa3.1 inhibitor, TRAM-34, was associated with a significant reduction in TGFβ1-activated pro-fibrotic gene expression, transdifferentiation of fibroblasts to myofibroblasts, and in wound healing in HCFs *in vitro*.

The TGFβ1 signaling proteins are key regulators of fibroblast differentiation to myofibroblasts which represents a major pathway in tissue fibrosis. Great efforts are under way in corneal research to understand the mechanisms of corneal fibrosis and identify pharmacological molecules [19], nanoparticles [9,17], or gene therapy approaches [16,18] to counterbalance TGFβ1 mediated pro-fibrotic function as a means of decreasing the myofibroblast population in injury-induced corneal fibrosis. The accumulating literature suggests that TRAM-34 significantly inhibits pro-fibrotic gene and protein expression by antagonizing TGFβ1 mediated downstream signaling, corroborating that KCa3.1 contributes to TGFβ1-driven fibrosis. Furthermore, *in vitro* studies show that TRAM-34 has the potential to exert inhibitory action on the fibrogenic properties of myofibroblasts [28]. The data findings of the current study build on the idea that of KCa3.1 blockade by TRAM-34 can control corneal fibrosis by limiting TGFβ1-induced pro-fibrotic functions (Fig 6).

KCa3.1 mediates the TGFβ1 induced cell proliferation and differentiation. Differentiation of fibroblasts to myofibroblasts symbolizes a key progression in tissue fibrogenesis, which involves the excessive expression of α-SMA, reorganization of actin cytoskeleton and incorporation of actin stress fibers [31]. A recent study in renal fibrosis utilizing knockout mouse model concluded that functional loss of KCa3.1 gene ameliorates the progression of fibrosis that leads to decreased fibroblast proliferation and collagen synthesis [12]. Our *in vivo* findings in the KCa3.1^-/-^ mice subjected to corneal injury confirm that attenuated corneal scarring occurs as a result of decreased myofibroblast differentiation, which translates to a decrease in haze formation. These results suggest that progression of corneal fibrosis is significantly blocked by loss of KCa3.1 channel activity. Blockade of the KCa3.1 function is known to negatively regulate the TGFβ1 signaling mediated downstream Smad pathways [14] involved in fibroblast cell proliferation. However, the mechanism by which KCa3.1 regulates cell proliferation and migration varies in different types of tissues. The major downstream signaling involved in KCa3.1 upregulation relies on the Ras/Raf/MEK/ERK- signaling pathway, independent of the p38 MAPK-signaling cascade [12, 32–34]. Apart from this, KCa3.1 is known to regulate JNK and C-Jun proteins in astrocytes [35]. In stem cells, KCa3.1 induces cycle progression via cyclins D1 and E [36]. These reports suggest that the KCa3.1 effects on cellular proliferation and migration are tissue specific.

Pharmacological inhibition of KCa3.1 is known to downregulate TGFβ1 dependent cell processes in normal and fibrotic tissues [13,14]. In this study, a marked decrease in TGFβ1 induced fibrotic marker expression was noted in corneal fibroblast cells treated by TRAM-34. Notably, a recent study in a renal fibrosis model showed that an increase in functional KCa3.1 expression causes increased cell proliferation and that pharmacological blockade of KCa3.1 by TRAM-34 targets cell cycle arrest in the G0/G1 phase, which prevents fibroblast proliferation in renal fibrosis [12]. These studies suggest that the KCa3.1 channel plays an intermediary role in TGFβ1 induced fibroblast cell proliferation and may provide a therapeutic approach for corneal fibrosis.

Currently, mitomycin C (MMC) is the drug of choice for the prevention of haze formation after surgical insult. While MMC has proven to effectively prevent postoperative corneal haze in some patients, its safety profile is controversial [37–39]. MMC treatment causes apoptosis, reduced proliferation, and sub-epithelial stromal loss. Administration of TRAM-34 is shown to prevent excessive cell proliferation *in vivo* and displays less toxic effect to cells than mitomycin [12, 28, 40]. Interestingly, a study of intra-ocular injection of TRAM-34 showed the beneficial effect in protecting ocular cells [41]. Our study further corroborates the effectiveness of TRAM-34 in blocking TGFβ induced mRNA and protein expression of myofibroblast markers. Our study validates the functional role of KCa3.1 in corneal fibrosis. The effectiveness of TRAM-34 in inhibiting cell proliferation while being associated with less toxicity to cells may make TRAM-34 a better therapeutic option for corneal fibrosis than currently available agents. Indeed, ICA-17043, a TRAM-34 analog, has been shown to be safe and has progressed to a Phase III clinical trial [42], suggesting that targeting the KCa3.1 channel may provide yet another therapeutic target for the prevention of corneal fibrosis. The findings related to pro-fibrotic gene expression in our transgenic KCa3.1 mouse model and the effects of pharmacological intervention with TRAM-34 show that KCa3.1 channels regulate fibrosis in the cornea *in vivo*. However, further TRAM-34 studies are warranted to optimize the methods and determine the dosing for the prevention of corneal fibrosis.

In conclusion, TRAM-34 has great potential to treat corneal fibrosis. The present study demonstrates that blockade of the KCa3.1 channel by TRAM-34 prevents injury-induced corneal fibrosis via inhibition of TGFβ -driven fibroblast migration and differentiation to myofibroblasts. TRAM-34 is likely to be a beneficial therapeutic agent to control injury-induced corneal fibrosis. Further studies are required to determine the *in vivo* application of TRAM-34 in corneal fibrosis.
